# Aktuelle Diagnostik und Therapie bei Ohrmuscheldysplasien und Gehörgangsfehlbildungen: Teil 1: Grundlagen und Diagnostik

**DOI:** 10.1007/s00106-023-01381-z

**Published:** 2023-11-03

**Authors:** Hannes H. Brandt, Daniel Bodmer

**Affiliations:** 1 Klinische Abteilung für Hals‑, Nasen‑, Ohrenkrankheiten, Universitätsklinikum St. Pölten, Dunant-Platz 1, 3100 St. Pölten, Österreich; 2 Hals-Nasen-Ohren-Klinik, Universitätsspital Basel, Basel, 4031 Petersgraben 4, Schweiz; 3 Karl Landsteiner Privatuniversität für Gesundheitswissenschaften, Krems, 3500 Dr. Karl-Dorrek-Straße 30, Österreich

**Keywords:** Kongenitale Anomalien, Rehabilitation, Cholesteatom, Hirnstammaudiometrie, Implantierbare Hörsysteme, Congenital abnormalities, Rehabilitation, Cholesteatoma, Evoked response audiometry, Implantable hearing aids

## Abstract

Angeborene Fehlbildungen der Ohrmuschel und des äußeren Gehörgangs können mit schweren ästhetischen und funktionellen Defiziten einhergehen. Neben dem Verständnis embryologischer Grundlagen ist auch die Klassifikation derartiger Anomalien für die Behandlung essenziell. Die frühzeitige Erkennung einer Fehlbildung sowie die Einleitung zeitgerechter Diagnostik sind essenziell, um durch eine adäquate Therapie langfristige Einschränkungen zu verhindern. Ohrmuscheldysplasien werden heute meist operativ, seltener mittels Epithese korrigiert. Die Methoden des „tissue engineering“ sind seit Langem in der Erprobung und könnten in Zukunft eine wesentliche Rolle spielen. Die Behandlung von Gehörgangsstenosen und -atresien bezweckt neben einer Hörverbesserung auch die Verhinderung von Folgeerkrankungen. Darunter finden sich u. a. Cholesteatome oder rezidivierende Infekte. Die Hörrehabilitation umfasst konventionelle und implantierbare Hörgeräte, wobei der Trend zu Letzteren tendiert.

## Abstract (en)

Congenital malformations of the pinna and aural atresia can result in major aesthetic and functional deficits. Knowledge about embryologic developments and established classification systems is an essential requirement when dealing with affected patients. Early detection of deficiencies and introduction of appropriate diagnostic measures is vital to initiate adequate therapies and prevent long-term disabilities. Treatment for malformations of the pinna—if requested—is mostly surgical, infrequently an epithesis is applied. As in other surgical fields, tissue engineering will likely play a crucial role in the future. Treatment of aural stenosis and atresia aims at improvement of hearing levels and prevention of secondary complications like cholesteatoma and chronic otorrhea. Auditory rehabilitation comprises a spectrum from conventional hearing aids to invasive hearing implants, the latter being favored in recent years.

## Lernziele

Die Behandlung angeborener Ohrmuscheldysplasien und Gehörgangsfehlbildungen ist in der HNO-ärztlichen Praxis selten. Die primäre Beurteilung erfolgt meist in größeren Zentren. Gewisse Dysplasien bzw. Defizite werden mitunter erst im Zuge der Früherkennungsuntersuchungen erkannt. Ein grundlegender Überblick der Thematik ist daher zur Einleitung der richtigen Schritte notwendig. Nach der Lektüre dieses Beitrags …

- kennen Sie die Epidemiologie und Pathogenese kongenitaler Fehlbildungen von Ohrmuschel und Gehörgang,
- können Sie die behandelnden Fehlbildungen mithilfe anerkannter Klassifizierungssysteme adäquat einteilen,
- haben Sie einen Überblick der für die Aufarbeitung notwendigen diagnostischen Schritte,
- wissen Sie, zu welchem Zeitpunkt die genannten diagnostischen Schritte indiziert sind,
- können Sie Eltern gezielt über das Ausmaß der Fehlbildung und den Umfang der Diagnostik informieren.

## Epidemiologie

Verschiedene epidemiologische Untersuchungen geben die Häufigkeit kongenitaler Ohrmuscheldysplasien im Bereich von 0,1–17,0/10.000 Geburten an [1, 2]. Lediglich bei einem Bruchteil davon handelt es sich um höhergradige Fehlbildungen. Die Häufigkeit von Gehörgangsfehlbildungen ist mit 0,5–1,3/10.000 Geburten etwas geringer [3, 4, 5]. Diese Daten sind aufgrund der hohen Variabilität der Datenqualität äußerst heterogen. Als Beispiel seien die diagnostischen Kriterien einer Dysplasie genannt. Es scheint jedoch eine geografische Prädisposition zu bestehen, da in den vorliegenden Erhebungen in Teilen Südamerikas bis zu 17 Fälle/10.000 Geburten gezählt wurden, während in Teilen Irlands nur eine Häufigkeit von 0,1/10.000 Geburten registriert wurde.

### Merke

Die Häufigkeit von Fehlbildungen des äußeren Ohrs liegt bei 0,1–17 Fällen pro 10.000 Geburten.

Eine Prädilektion beider Fehlbildungen wird in der Literatur für das rechte Ohr und das **männliche Geschlecht**

Männliches Geschlecht

beschrieben. Im Fall einer kombinierten Fehlbildung entspricht das Ausmaß der Dysplasie meist dem der Atresie. Höhergradige Ohrmuscheldysplasien gehen zumeist (in 55–93 % der Fälle) mit einer Gehörgangsatresie oder -stenose einher. Bilaterale Gehörgangsdysplasien treten in 10 % der Fälle auf [2, 6, 7].

Ohrmuscheldysplasien und Gehörgangsfehlbildungen entstehen zu 80–90 % aus **sporadischen Mutationen**

Sporadische Mutationen,

5 % sind vererbt, 10 % stehen mit Syndromen in Zusammenhang (Goldenhar, Treacher-Collins, hemifaziale Mikrosomie) [8]. Eine Assoziation mit intrauterinen **toxischen Einflüssen**

Toxische Einflüsse

(Vitamin A, Methamphetamin, Alkohol) wurde beschrieben [9]. Zudem wurde ein Zusammenhang mit **intrauteriner Hypoxie**

Intrauterine Hypoxie

bei Bewohnern in Höhenlagen postuliert [2].

Eine vollständige Gehörgangsatresie ist je nach Autor 7‑ bis 12-fach häufiger als eine Gehörgangsstenose [10, 11].

### Merke

Schwere Ohrmuscheldysplasien sind selten und bringen ein hohes Risiko einer assoziierten Gehörgangsanomalie mit sich.

## Definition und Klassifikation

Unter einer Ohrmuscheldysplasie wird eine in Form und Größe aberrant entwickelte Ohrmuschel verstanden. Eine allgemein anerkannte Definition dieses Begriffs existiert nicht. Im englischen Sprachgebrauch wird für Ohrmuscheldysplasien höheren Grades gerne das Wort „microtia“ verwendet. Nicht alle derartigen Fehlbildungen gehen jedoch mit einer geringeren Größe einher. Zudem handelt es sich, entsprechend der unten angeführten Klassifikation von Weerda, auch bei der Makrotie um eine Ohrmuscheldysplasie. Der englische Terminus kann daher verwirrend sein.

### Merke

Eine weltweit einheitliche Nomenklatur für die Klassifikation von Ohrmuscheldysplasien und Gehörgangsanomalien existiert nicht.

Ohrmuscheldysplasien wurden erstmals 1926 durch Marx in 3 verschiedene Grade eingeteilt [12]. Es folgten über die Jahrzehnte Einteilungen unterschiedlicher Komplexität. In einem internationalen Positionspapier aus dem Jahr 2019 wird die Klassifikation von Weerda als „breit angewandt“ bewertet [13, 14]. In Tab. 1 findet sich eine Übersicht dieser beiden Klassifikationen. Zusammenfassend ist anzumerken, dass bei Anlage sämtlicher Landmarken eine Ohrmuscheldysplasie Grad 1 vorliegt. Hier sind beispielhaft eine **Apostasis otis**

**Figure 1:**
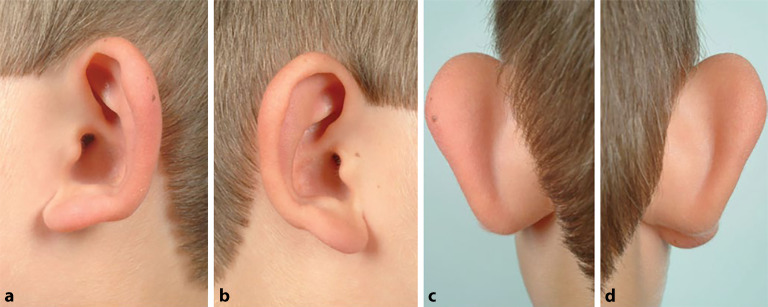

**Figure 2:**
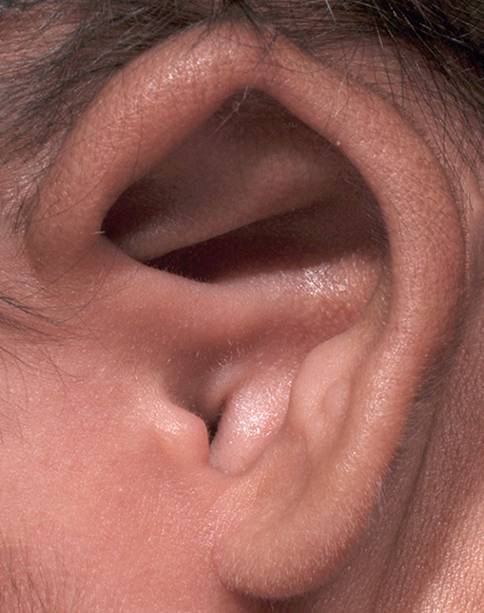

**Figure 3:**
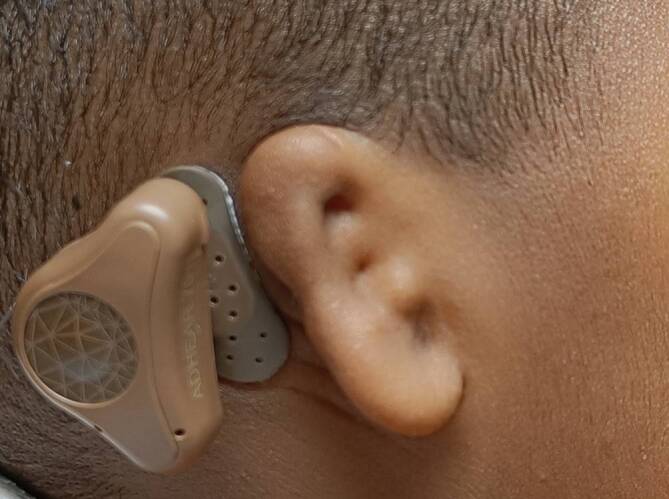

**Figure 4:**
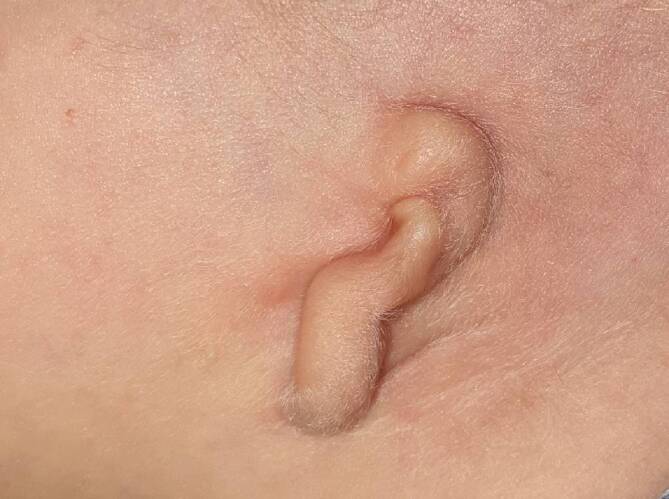

Apostasis otis

(Abb. 1) oder Aberrationen im Bereich des Crus helicis (Abb. 2) zu nennen. Bei höhergradigen Dysplasien fehlen die klassischen Landmarken teilweise (Abb. 3) oder vollständig (Abb. 4).

**Table 1:** 

Weerda (1988) [14]	Marx (1926) [12], modifiziert nach Rogers (1977)
*Dysplasie I. Grades:* Die meisten Strukturen einer normalen Ohrmuschel sind vorhanden. A Makrotie B Apostasis otis (Abstehohren) C Kryptotie (Taschenohr) D fehlende Helixausformung E leichte Fehlbildungen von Tragus und Lobulus F Kolobom/Satyrohr/Stahlohr G Lobulushyperplasie/-hypoplasie H Tassenohrdeformität Typ I + II	*Grad I:* abnormale Ohrmuschel, alle Landmarken sind identifizierbar
*Dysplasie II. Grades:* Manche Strukturen einer normalen Ohrmuschel sind vorhanden. A Tassenohrdeformität Typ III B „Miniohren“	*Grad II:* abnormale Ohrmuschel, manche Landmarken sind nicht mehr identifizierbar
	*Grad III:* kleines Ohranhängsel
*Dysplasie III. Grades:* Keine Strukturen einer normalen Ohrmuschel sind vorhanden. A unilateral B bilateral C Anotie	*Grad IV:* Anotie

Gehörgangsatresien werden als eine **fehlende Verbindung**

Fehlende Verbindung

zwischen dem Gehörgangseingang und dem Mittelohr definiert. Assoziierte Strukturen wie die **Ossikelkette**

Ossikelkette

oder der N. facialis können in unterschiedlichem Maße zusätzlich betroffen sein. Von der Gehörgangsatresie ist die Gehörgangsstenose abzugrenzen. Letztere wurde durch Cole und Jahrsdoerfer durch ein Gehörgangslumen <4 mm definiert, da in diesem Fall ein signifikant **erhöhtes Cholesteatomrisiko**

**Figure 5:**
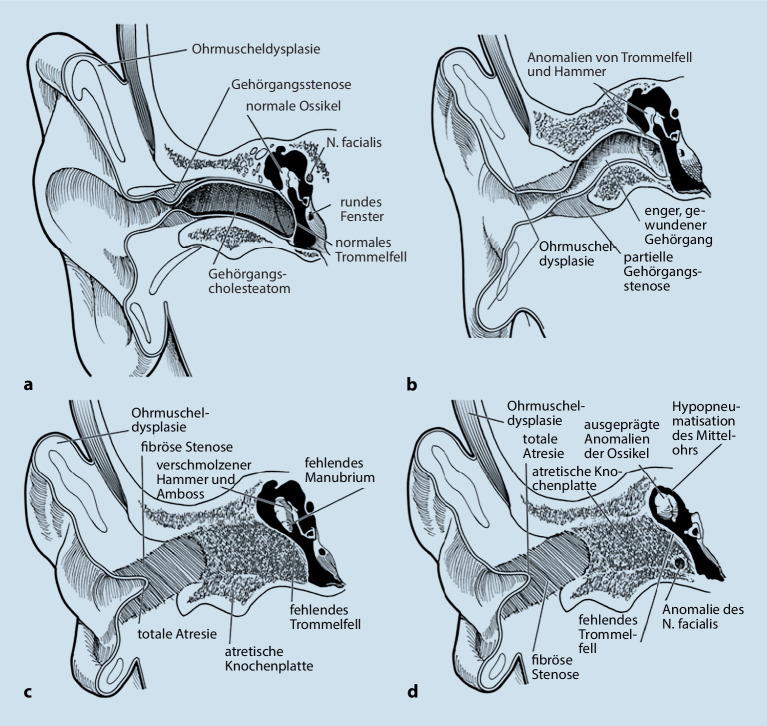

Erhöhtes Cholesteatomrisiko

besteht [10]. Dieses wird gemäß einer Analyse von Casale mit etwa 20 % angegeben [16]. Die gebräuchlichsten Klassifikationen der Gehörgangsdysplasien sind in Tab. 2 zusammengefasst, Abb. 5 zeigt die Klassifikation nach Schuknecht [17, 18, 19]. Die Klassifikationen sind für akademische Zwecke geeignet, bieten jedoch oft keine optimale klinische Anwendbarkeit. So setzen manche Klassifikationen der Gehörgangsatresie bereits Kenntnisse über die Beschaffenheit von Mittelohrstrukturen oder die Art der Atresie voraus. Diese sind in einer klinischen Untersuchung nicht erhebbar. Hierfür ist zunächst eine Abklärung mittels hochauflösender **Computertomographie**

Computertomographie

(CT) des Felsenbeins nötig, die meistens bis in das Vorschulalter aufgeschoben wird. In einer gängigen Untersuchung kann jedoch eine einfache Einteilung des Gehörgangs in „normal“, „stenotisch“, „blind endend“ oder „atretisch“ vorgenommen werden.

**Table 2:** 

Weerda (1994) [17]	Schuknecht (1989) [20], Abb. 5	Altmann (1955) [19]
*Gehörgangsstenose Typ A*: starke Einengung des Gehörgangs mit intaktem Hautschlauch	*Typ a:* häutige/knorpelige Gehörgangsstenose im lateralen Anteil des äußeren Gehörgangs	*Typ I:* kleiner Gehörgang; hypoplastisches Os temporale und Trommelfell; normal angelegtes/hypoplastisches Tympanon; normal angelegte oder dysplastische Ossikel
*Gehörgangsstenose Typ B*: teilweise angelegter Gehörgang mit einer Atresieplatte im medialen Anteil	*Typ b:* Stenose sowohl im knorpeligen wie auch im knöchernen Teil des äußeren Gehörgangs; Größe des Trommelfells oft reduziert bzw. teilweise durch ein knöchernes Septum ersetzt; Ossikeldysplasien in der Paukenhöhle	*Typ II:* kein durchgängiger Gehörgang (mit Atresieplatte); hypoplastisches Tympanon; dysplastischer und fixierter Malleolus und Incus
*Gehörgangsatresie Typ C*: komplette knöcherne Gehörgangsatresie	*Typ c:* vollständige Gehörgangsatresie mit gut entwickelter Belüftung der Paukenhöhle; kein Trommelfell vorhanden, Hammerkopfdysplasie und -fixation an der Atresieplatte; aberranter Fazialisverlauf (Verlagerung nach anterior)	*Typ III:* kein durchgängiger Gehörgang; hypoplastisches oder fehlendes Tympanon mit oder ohne Ossikel
	*Typ d:* zusätzlich zu Typ c hypopneumatisierte Paukenhöhle; Dysplasien des knöchernen Labyrinths

### Merke

Ohrmuscheldysplasien und Gehörgangsanomalien lassen sich durch unterschiedliche Klassifikationen in verschiedene Schweregrade einteilen.

## Embryologie

Sowohl das **äußere Ohr**

Äußeres Ohr

– bestehend aus Ohrmuschel und Gehörgang – als auch das **Mittelohr**

Mittelohr

entstammen den ersten beiden **Kiemenbögen**

Kiemenbögen.

Die Entwicklung des Innenohrs ist davon unabhängig, weshalb selten kombinierte Dysplasien vorliegen. Die hier behandelten kongenitalen Dysgenesien werden durch Fehler in der embryonalen Entwicklung verursacht. Eine kurze Zusammenfassung der verschiedenen Schritte soll das Verständnis erleichtern.

### Merke

Die Innenohrentwicklung ist unabhängig von der Entwicklung des äußeren und des Mittelohrs, daher treten gleichzeitige Anlagestörungen des Innenohrs nur selten auf.

Die Ohrmuschel entsteht zwischen der 4. und 12. Gestationswoche aus **mesenchymalem Gewebe**

**Figure 6:**
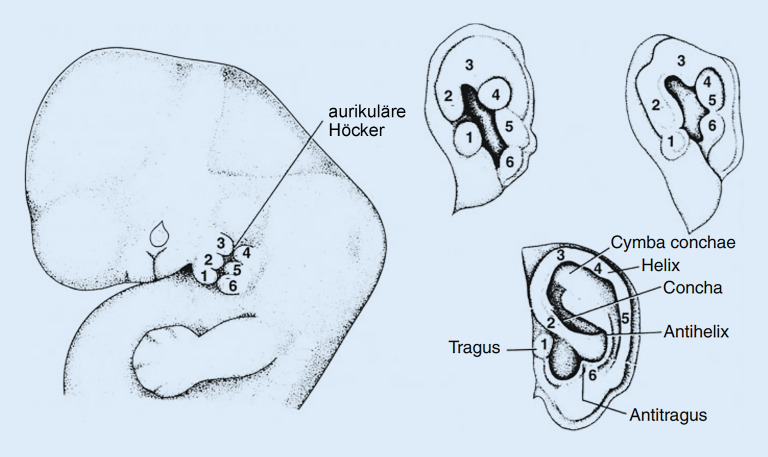

Mesenchymales Gewebe

des ersten und zweiten Kiemenbogens – den 6 Höckern von His (Abb. 6). Aus den ersten 3 Höckern entwickeln sich dabei der Tragus und die Crus helicis, aus den Höckern 4–6 der Lobulus, die Helix und die Anthelix. Eine sorgfältige Analyse einer Ohrmuschelfehlbildung lässt Rückschlüsse auf die beteiligten Höcker zu. Diesbezüglich sei auf eine Übersichtsarbeit von Bartels verwiesen [21]. Durch den Grad der Ohrmuscheldysplasie können Rückschlüsse auf den Zeitpunkt der Fehlentwicklung gezogen und somit andere Defizite vorausgesagt werden [7]. Bei Geburt weisen normal entwickelte Ohren etwa 2 Drittel ihrer endgültigen kraniokaudalen Länge und 76 % ihrer endgültigen anteroposterioren Breite auf [22].

Der Gehörgang entwickelt sich zusammen mit dem Antrum mastoideum und dem epithelialen Anteil des Trommelfells ab der 8. Gestationswoche aus der 1. Kiemenfurche. Aus einem zunächst soliden Areal entsteht ab der 28. Woche zunächst medial, dann lateral das spätere Gehörgangslumen [24]. Sollte dieser Prozess unvollständig ablaufen, entsteht eine Gehörgangsstenose oder -atresie.

### Merke

Die (Fehl‑)Entwicklung der Ohrmuschel findet zwischen der 4. und 12. Gestationswoche statt, die der Gehörgangsanlage zwischen der 28. und 30. Woche.

## Diagnostik

Fehlbildungen des äußeren Ohrs werden in der Regel kurz nach der Geburt bemerkt, wenn im Rahmen der **verschiedenen Screenings**

Verschiedene Screenings

(Neugeborenen-Hörscreening) eine Anomalie im Bereich der Ohrmuschel oder ein nicht angelegter Gehörgang auffallen. Von diesem Zeitpunkt an ist es wichtig, die notwendigen Abklärungen fristgerecht durchzuführen und weitere Untersuchungen korrekt zu priorisieren. Isolierte Fehlbildungen von Gehörgang oder Mittelohr bei normal entwickelter Ohrmuschel können eine verzögerte Diagnosestellung zur Folge haben.

Die Beurteilung durch **Humangenetiker**

Humangenetiker

kann dabei helfen, ein möglicherweise vorliegendes **syndromales Krankheitsbild**

Syndromales Krankheitsbild

zu diagnostizieren.

Die enge Assoziation von Ohrmuschel- und Gehörgangsfehlbildungen wurde bereits beschrieben. Die weiteren diagnostischen Schritte ähneln sich zumeist und sollen daher im Folgenden gemeinsam besprochen werden.

Einer klinischen Untersuchung schließt sich bei nicht bestandenem **Neugeborenen-Hörscreening**

Neugeborenen-Hörscreening

in der Regel eine **Hirnstammaudiometrie**

Hirnstammaudiometrie

mit separater Ableitung der Schwellen für die Luft- und Knochenleitung an (Infobox). Auf diese Weise kann die Hörleistung des Innenohrs sowie eine eventuelle Schallleitungsschwerhörigkeit quantifiziert werden. Die Messung sollte auch bei einseitigen Dysplasien stets beidseits erfolgen, um eine in 10–25 % der Fälle vorliegende Hörminderung der Gegenseite auszuschließen [7]. Eine vollständige Gehörgangsatresie geht in der Regel mit einer Air-Bone-Gap von 45–60 dB HL einher. Hörschwellen über 70 dB HL machen eine zusätzliche sensorineurale Komponente wahrscheinlich.

### Merke

Offensichtliche Fehlbildungen des Ohrs fallen meist bei Neugeborenenscreenings auf. Die Hirnstammaudiometrie ist oft der nächsten Abklärungsschritt.

Eine weiterführende radiologische Abklärung mittels Feinschicht-CT des Felsenbeins kann aufgrund mangelnder Konsequenz bis zum Alter von 4–6 Jahren zurückgestellt werden [7]. Hierdurch ist eine Einschätzung bezüglich der Ausprägung sowie eine endgültige Einordnung der Fehlbildung von Gehörgang und Mittelohrstrukturen möglich. Eine entsprechende Diagnostik sollte nur dann durchgeführt werden, wenn daraus eine entsprechende Konsequenz resultiert. Durch die Kenntnis eingangs dargestellter embryologischer Entwicklung kann eine **Schichtbildgebung**

Schichtbildgebung

in bestimmten Fällen vermieden werden. Indiziert ist eine solche Untersuchung bei **Schallleitungsblöcken**

**Figure 7:**
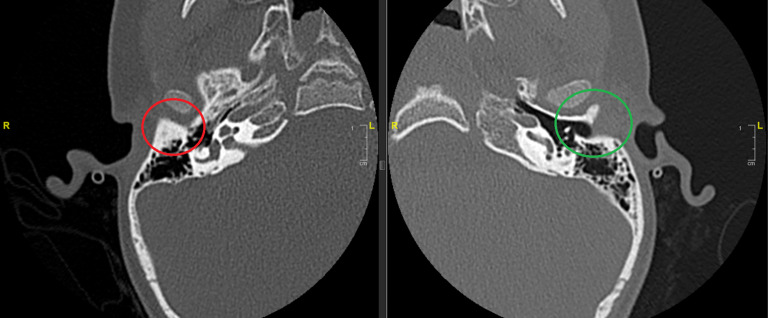

Schallleitungsblöcke

unklarer Genese oder klinisch diagnostizierten Gehörgangsatresien (Abb. 7).

### Merke

Eine Abklärung mittels CT sollte nur bei einer unmittelbaren therapeutischen Konsequenz durchgeführt werden und kann oft bis ins Vorschulalter warten.

Die nachgewiesenen Dysplasien treten dabei unterschiedlich häufig auf. In etwa der Hälfte der Fälle findet sich bei Patienten mit höhergradigen Ohrmuscheldysplasien oder Gehörgangsatresien ein **verminderte Mastoidpneumatisation**

Verminderte Mastoidpneumatisation,

in fast 90 % der Fälle ist hier auch das Volumen der Pauke reduziert [25]. **Ossikeldysplasien**

**Figure 8:**
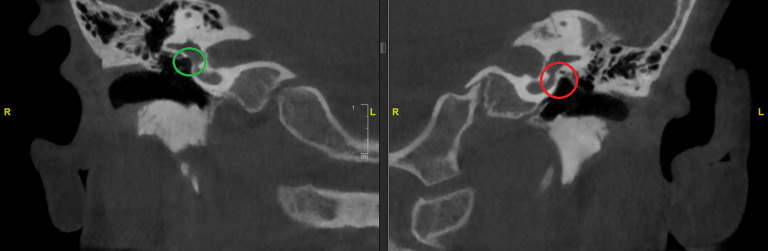

Ossikeldysplasien

sind ebenfalls häufig. Oft zeigt sich ein dysplastisch verklumpter Hammer-Amboss-Komplex oder nur rudimentär angelegte Ossikelanteile. Der Stapes wird von allen Ossikeln (mit 31 % bei höhergradigen Fehlbildungen) noch am häufigsten angetroffen. Das runde Fenster scheint in 90 % der Fälle offen, während das ovale Fenster bei 40 % der Patienten mit Gehörgangsatresien und höhergradigen Ohrmuscheldysplasien verschlossen ist (Abb. 8). Der Verlauf des **N. facialis**

**Figure 9:**
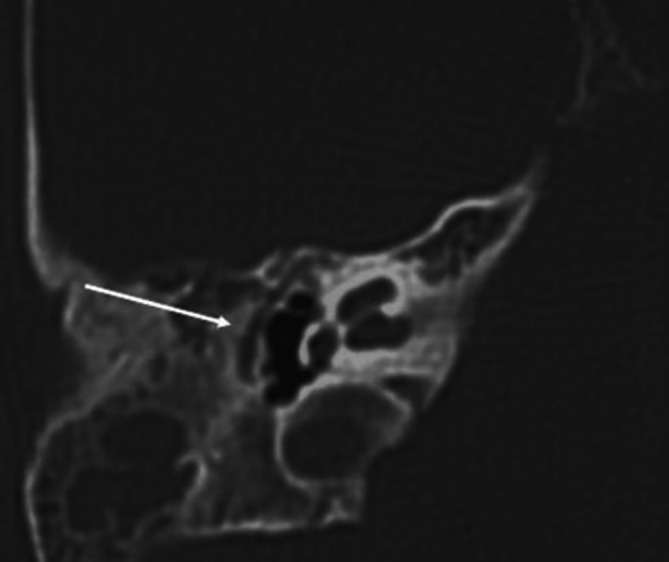

N. facialis

ist bei mehr als 3 Vierteln der Patienten mit höhergradigen Dysplasien verändert. Im Mittelohr wird dabei oft eine Verlagerung nach kaudal, im Mastoid hingegen nach ventral beobachtet (Abb. 9). Der Austritt aus der Schädelbasis ist im Rahmen einer Gehörgangsatresie häufig weiter kranial. Entsprechende Kenntnisse sind zur Vermeidung intraoperativer Komplikationen von höchster Wichtigkeit. Assoziierte Fehlbildungen des Innenohrs wurden bis auf diskrete Veränderungen des lateralen Bogengangs in einer Analyse von Siegert et al. nicht beobachtet [25]. Diesbezüglich wird erneut auf die Bemerkungen im Abschnitt „Embryologie“ hingewiesen.

Zur Abschätzung des Erfolgs einer operativen Therapie gibt es **verschiedene Punktesysteme**

Verschiedene Punktesysteme,

welche in Tab. 3 und 4 dargestellt sind [11, 25]. Mithilfe dieser Einteilungen werden die anatomischen Verhältnisse der dysplastischen Gehörgangs und Mittelohrs beurteilt. Bei niedrigen Werten empfehlen die Autoren, auf eine Rekonstruktion des atretischen Gehörgangs zu verzichten, da in diesen Fällen keine realistische Chance auf eine signifikante Besserung des Hörvermögens besteht.

**Table 3:** 

Struktur	Konfiguration	Punkte
Meatus acusticus externus	Normal	2
	Weichgewebsatresie	1
	Knochenatresie	0
Mastoidbelüftung	Ausgeprägt	2
	Mittelgradig	1
	Keine	0
Paukengröße	Groß	2
	Mittelgradig	1
	Eburnisiert	0
Paukenbelüftung	Ausgeprägt	2
	Mittelgradig	1
	Keine	0
N. facialis	Normal	4
	Gering disloziert	2
	Stark disloziert	0
Gefäßverläufe	Normal	2
	Gering disloziert	1
	Stark disloziert	0
Hammer und Amboss	Normal	2
	Dysplastisch	1
	Fehlt	0
Stapes	Normal	4
	Dysplastisch	2
	Fehlt	0
Ovales Fenster	Offen	4
	Verschlossen	0
Rundes Fenster	Offen	4
	Verschlossen	0
*Punktsumme: 0–28* Bei beidseitiger Fehlbildung wurde eine Mittelohrrekonstruktion auf dem besser hörenden Ohr ab 15 Punkten angeboten. Bei einseitiger Fehlbildung wurde nach kritischer Diskussion mit dem Patienten ab 20 Punkten eine Mittelohrrekonstruktion angeboten. Bei geringeren Punktwerten wurde ausschließlich eine Hörgeräteversorgung empfohlen

**Table 4:** 

Nachweisbare Struktur in hochauflösender Computertomographie (CT) des Felsenbeins	Punkte
Stapes	2
Ovales Fenster	1
(Lufthaltiges) Tympanon	1
Fazialisnerv	1
Malleus-Incus-Komplex	1
Pneumatisiertes Mastoid	1
Incus-Stapes-Verbindung	1
Rundes Fenster	1
Äußerer Gehörgang nachweisbar	1

Ab einem Wert >/≥ 6 (je nach Autor) ist ein chirurgischer Ansatz empfehlenswert und mit einer signifikanten Verbesserung der postoperativen Hörleistung assoziiert [27]

## Planung von Diagnostik und Therapie

Grundsätzlich sollte die Diagnostik und Therapie an die individuellen Bedürfnisse der Patienten angepasst werden. Im Vorfeld kann man so auch auf die **unterschiedlichen Erwartungshaltungen**

Unterschiedliche Erwartungshaltungen

eingehen. Zum Überblick über die verschiedenen diagnostischen Maßnahmen kann ein **Flussdiagramm**

**Figure 10:**
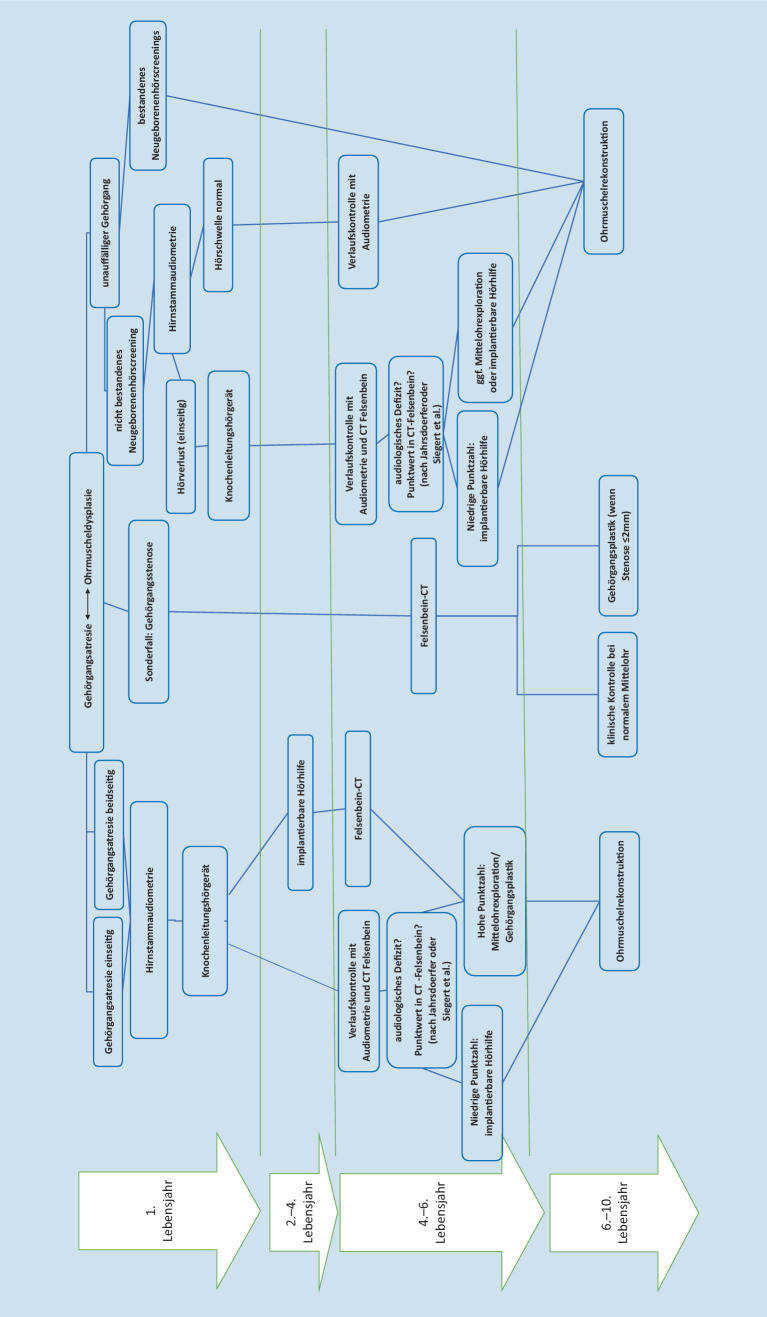

Flussdiagramm

hilfreich sein. Frenzel et al. entwarfen hierfür in der Vergangenheit das „Lübeck Flowchart for Functional and Aesthetic Rehabilitation of Aural Atresia and Microtia“ [28]. Dieses diente auch als Inspiration für eine etwas detailliertere Fassung (Abb. 10).

Die angesprochenen therapeutischen Maßnahmen werden im zweiten Teil dieser Fortbildung besprochen.

### Infobox Hirnstammaudiometrie

Die Hirnstammaudiometrie ist ein objektives audiometrisches Verfahren, bei dem durch auditorische Stimuli verursachte neuronale Ströme entlang der Hörbahn mittels Oberflächenelektroden an der Kopfhaut abgeleitet werden. Voraussetzung für verwertbare Ergebnisse ist eine Ableitung in geräuscharmer Umgebung in körperlicher Ruhe. Während dies bei Erwachsenen im Wachzustand erfolgt, wird die Messung bei Säuglingen und Kleinkindern nach Möglichkeit im Spontanschlaf durchgeführt. Mit den Eltern kann zur idealen Vorbereitung eine verkürzte Schlafperiode in der Nacht oder am Tag vor der Untersuchung besprochen werden. Sofern die Maßnahme von den jungen Patienten nicht toleriert wird, kann eine Allgemeinanästhesie oder Sedierung notwendig werden.

## Fazit für die Praxis

- Höhergradige Ohrmuschelfehlbildungen und Gehörgangsfehlbildungen sind seltene Krankheitsbilder.
- Eine frühe Diagnose ist wichtig, um die notwendigen Therapien einzuleiten.
- Während die (Fehl‑)Bildung der Ohrmuschel embryologisch zwischen der 4. und 12. Gestationswoche geschieht, erfolgt eine (fehlende) Kanalisierung des Gehörgangs erst in der 28. Schwangerschaftswoche.
- Unter den zahlreichen Klassifikationssystemen für Ohrmuscheldysplasien ist die Einteilung nach Weerda weit verbreitet.
- Für die Einteilung der Gehörgangsatresien existieren ebenfalls mehrere Systeme.
- Zu Beginn einer diagnostischen Aufarbeitung steht meist eine Hirnstammaudiometrie.
- Eine hochauflösende Computertomographie des Felsenbeins stellt insbesondere zur Einordnung einer Gehörgangsfehlbildung, einer möglicherweise assoziierten Mittelohrdysplasie und zur Abschätzung des Erfolgs einer möglichen operativen Rekonstruktion einen wichtigen diagnostischen Eckpfeiler dar – die Indikation ist kritisch zu stellen.
- Die Eltern sollten grundsätzlich in einem oft über viele Jahre andauernden Behandlungsprozess von Anfang an umfassend über die gestaffelten Schritte der Diagnostik und später der Therapie orientiert werden.

## CME-Fragebogen

Welche Aussage zu Epidemiologie und den Risikofaktoren kongenitaler Fehlbildungen von Ohrmuschel und Gehörgang trifft zu?

Beide Ohren sind in etwa gleich häufig von Ohrmuscheldysplasie und Gehörgangsfehlbildungen betroffen.

Gehörgangsatresien sind in etwa 10-mal häufiger als Ohrmuscheldysplasien.

Mütterlicher Alkoholkonsum in der Schwangerschaft erhöht das Risiko für eine Ohrmuscheldysplasie.

Gehörgangsatresien und -stenosen kommen in der Regel im Rahmen verschiedener Fehlbildungssyndrome vor, sporadische Mutationen sind mit 10 % eher selten.

Gehörgangsstenosen kommen häufiger vor als Gehörgangsatresien.

Welche Aussage zur Embryologie der Ohrmuschel- und Gehörgangsentwicklung ist richtig?

Die kraniokaudale Länge der Ohrmuschel verdoppelt sich normalerweise zwischen der Geburt und dem 18. Lebensjahr.

Weibliche Neugeborene leiden seltener an Fehlentwicklungen der Ohrmuschel als männliche.

Die Ohrmuschel entwickelt sich aus insgesamt 8 Höckern der ersten beiden Kiemenbögen.

Der äußere Teil des Trommelfells entsteht aus der zweiten Kiemenfurche.

Die Kanalisierung des Gehörgangs und die Ausformung der Anthelixfalte finden während der embryologischen Entwicklung in etwa gleichzeitig statt.

In Ihrer Sprechstunde wird eine Familie vorstellig, deren mittlerweile 3 Wochen alter Sohn das Hörscreening für Neugeborene links nicht bestanden hat. Zudem wurde kurz nach der Geburt bereits eine höhergradige Ohrmuscheldysplasie diagnostiziert (Abb. 4). Die Eltern wünschen eine möglichst zeitnahe Korrektur der Ohrmuschel. Welche Aussage zur weiteren Therapie ist richtig?

Primäre Anfertigung einer Computertomographie (CT) des Felsenbeins, um die Durchgängigkeit des Gehörgangs sowie das Vorhandensein von Ossikeln und Cochlea zu verifizieren.

Eine Rekonstruktion der Ohrmuschel ist innerhalb von 12–24 Monaten anzustreben.

Die Durchgängigkeit des Gehörgangs ist mit hoher Wahrscheinlichkeit gegeben.

Es handelt sich um eine Ohrmuscheldysplasie Grad II nach Weerda.

Zeitnah sollte eine Hirnstammaudiometrie erfolgen, um das Hörvermögen auf dem betroffenen Ohr zu prüfen.

Die Eltern eines einmonatigen Säuglings kommen zur Konsultation und Planung weiterer diagnostischer Schritte in Ihre Sprechstunde. Wenige Tage nach der Geburt war den Eltern eine dysplastische linke Ohrmuschel aufgefallen, welche durch das Fehlen klassischer Landmarken charakterisiert ist. Was trifft zur weiteren Abklärung bei dem genannten Patienten zu?

Das Risiko einer Gehörgangsdysplasie ist bei Patienten mit drittgradiger Ohrmuscheldysplasie im Vergleich zu Patienten mit einseitiger Apostasis otis nicht signifikant erhöht.

Auch bei sondierbarem Gehörgang und einsehbarem Trommelfell sollte bei genanntem Patienten zeitnah eine Bildgebung mittels Computertomographie (CT) des Felsenbeins erfolgen, um eine Mittelohrfehlbildung auszuschließen.

Sofern in genanntem Fall klinisch eine knöcherne Gehörgangsatresie nachgewiesen wird, ergibt sich hieraus eine direkte operative Behandlungsindikation.

Bei unauffälligem Gehörgangsbefund und normwertigen Hörschwellen in der Hirnstammaudiometrie kann auf eine CT des Felsenbeins verzichtet werden.

Die ästhetische Korrektur der genannten Ohrmuscheldysplasie sollte vor einer eventuellen Hörrehabilitation erfolgen.

Sie beraten eine Familie, welche für ihre 4‑jährige Tochter eine operative Korrektur bei einer Tassenohrdeformität wünscht. Welche Aussage zur Entwicklung der Ohrmuschel ist richtig?

Die Ohrmuschel hat entodermale und mesenchymale Anteile.

Die Concha der Ohrmuschel entsteht aus den Höckern 1 und 2.

Fehlentwicklungen von Ohrmuschel und Gehörgang entwickeln sich unabhängig von Innenohrmissbildungen.

Die Tassenohrdeformität ist auf eine Aplasie der Höcker 1 und 6 zurückzuführen.

Die Tassenohrdeformität I und II werden nach Weerda als Ohrmuscheldysplasien zweiten Grades eingeordnet.

Nach nicht bestandenem Neugeborenenscreening stellt sich eine junge Familie zur Durchführung einer pädaudiologischen Abklärung vor. Bei weiterhin nicht reproduzierbaren otoakustischen Emissionen entscheiden Sie sich zur Durchführung einer Hirnstammaudiometrie. Welche Aussage trifft zu?

Für eine Hirnstammaudiometrie ist zumindest eine Sedation, zur besseren Aussagekraft jedoch meist eine Allgemeinanästhesie nötig.

Die jungen Patienten sollten ausgeschlafen zur Untersuchung erscheinen.

Bei der Hirnstammaudiometrie handelt es sich um ein subjektives audiometrisches Verfahren.

Es kann eine separate Messung der Hörschwellen für Luft- und Knochenleitung erfolgen.

Da die Ableitungselektroden unter die Haut platziert werden, handelt es sich um eine invasive diagnostische Maßnahme.

Welche Antwort trifft bezüglich der zeitlichen Einordnung von Diagnostik und Therapie bei Gehörgangsatresien und Ohrmuscheldysplasien zu?

Ein nicht bestandenes Neugeborenen-Hörscreening sollte im Alter von 2–3 Jahren mittels Reaktionsaudiometrie kontrolliert werden.

Wird eine Ohrmuscheldysplasie im Vorschulalter nicht versorgt, verspricht ein Eingriff zu einem späteren Zeitpunkt im Leben keinen signifikanten ästhetischen Erfolg.

Bei einseitiger Gehörgangsatresie besteht innerhalb des ersten Lebensjahres die Indikation zur weiteren Abklärung mittels Feinschicht-Computertomographie des Felsenbeins.

Die Hirnstammaudiometrie spielt in der frühen Diagnostik (1. Lebensjahr) bei Gehörgangsatresien eine untergeordnete Rolle.

Auch bei normal angelegtem Mittelohr und unauffälligem Gehör besteht bei einer Gehörgangsstenose < 2 mm eine Behandlungsindikation.

Sie haben bei einem 4‑jährigen Patienten mit klinisch diagnostizierter Gehörgangsatresie zur Planung der weiteren Therapie eine Feinschicht-Computertomographie des Felsenbeins durchführen lassen. Welche Aussage zu den dort erhobenen Befunden trifft zu?

Gehörgangsfehlbildungen sind in der Regel mit Fehlbildungen des Innenohrs assoziiert.

Sofern eine Ossikeldysplasie vorliegt, ist der Stapes am häufigsten betroffen.

Auch bei höhergradigen Dysplasien ist das runde Fenster in etwa 90 % der Fälle darstellbar und offen.

Aberrante Verläufe des N. facialis im Rahmen einer Dysplasie von Gehörgang und Mittelohr sind selten.

Das Mastoid ist bei Patienten mit Gehörgangsatresien meist normal pneumatisiert.

Für die Bewertungen einer Computertomographie des Felsenbeins nach dem Jahrsdoerfer Grading System und dem Punktesystem von Siegert et al. gilt:

Die maximal erreichbare Punktzahl bei beiden Systemen ist 10.

Ein wesentlicher Unterschied des Punktesystems nach Siegert et al. liegt in der Berücksichtigung des Vorliegens einer Dysplasie an einem oder beiden Ohren.

Je niedriger der Wert in beiden Systemen ausfällt, desto eher sollte eine operative Rekonstruktion einer Gehörgangsatresie/Mittelohrdysplasie erfolgen.

Die Pneumatisation des Mastoids hat auf die Punktwerte beider Systeme keinen Einfluss.

Für eine volle Punktzahl in beiden Systemen muss eine Cochlea mit 2,5 Windungen computertomographisch abgrenzbar sein.

Bezüglich der Klassifikationssysteme von Ohrmuscheldysplasien und Gehörgangsfehlbildungen trifft folgende Aussage zu:

Der englische Terminus „microtia“ beschreibt eine Ohrmuscheldysplasie Grad II nach Weerda.

Die Klassifikation nach Weerda für Gehörgangsfehlbildungen setzt eine Computertomographie-Bildgebung des Felsenbeins voraus.

Die Einteilung der Gehörgangsfehlbildungen nach Schuknecht schließt in der Beurteilung ebenfalls Mittelohrstrukturen ein.

Das Vorhandensein von Landmarken wie dem Tragus spielt für die Einordnung einer Ohrmuscheldysplasie keine Rolle.

Die Klassifikation nach Weerda für Ohrmuscheldysplasien ist international ohne Alternative.
